# Local adaptation and spatiotemporal patterns of genetic diversity revealed by repeated sampling of *Caenorhabditis elegans* across the Hawaiian Islands

**DOI:** 10.1111/mec.16400

**Published:** 2022-02-25

**Authors:** Timothy A. Crombie, Paul Battlay, Robyn E. Tanny, Kathryn S. Evans, Claire M. Buchanan, Daniel E. Cook, Clayton M. Dilks, Loraina A. Stinson, Stefan Zdraljevic, Gaotian Zhang, Nicole M. Roberto, Daehan Lee, Michael Ailion, Kathryn A. Hodgins, Erik C. Andersen

**Affiliations:** ^1^ 3270 Department of Molecular Biosciences Northwestern University Evanston Illinois USA; ^2^ 2541 School of Biological Sciences Monash University Melbourne Victoria Australia; ^3^ 3270 Interdisciplinary Biological Sciences Program Northwestern University Evanston Illinois USA; ^4^ 7284 Department of Biochemistry University of Washington Seattle Washington USA

**Keywords:** *Caenorhabditis*, *Caenorhabditis elegans*, genetic diversity, local adaptation, niche

## Abstract

The nematode *Caenorhabditis elegans* is among the most widely studied organisms, but relatively little is known about its natural ecology. Genetic diversity is low across much of the globe but high in the Hawaiian Islands and across the Pacific Rim. To characterize the niche and genetic diversity of *C. elegans* on the Hawaiian Islands and to explore how genetic diversity might be influenced by local adaptation, we repeatedly sampled nematodes over a three‐year period, measured various environmental parameters at each sampling site, and whole‐genome sequenced the *C. elegans* isolates that we identified. We found that the typical Hawaiian *C. elegans* niche comprises moderately moist native forests at high elevations (500–1,500 m) where ambient air temperatures are cool (15–20°C). Compared to other *Caenorhabditis* species found on the Hawaiian Islands (*e.g*., *Caenorhabditis briggsae* and *Caenorhabditis tropicalis*), we found that *C. elegans* were enriched in native habitats. We measured levels of genetic diversity and differentiation among Hawaiian *C. elegans* and found evidence of seven genetically distinct groups distributed across the islands. Then, we scanned these genomes for signatures of local adaptation and identified 18 distinct regions that overlap with hyper‐divergent regions, which may be maintained by balancing selection and are enriched for genes related to environmental sensing, xenobiotic detoxification, and pathogen resistance. These results provide strong evidence of local adaptation among Hawaiian *C. elegans* and contribute to our understanding of the forces that shape genetic diversity on the most remote volcanic archipelago in the world.

## 1 INTRODUCTION

The nematode *Caenorhabditis elegans* is a powerful model organism that has facilitated advances in the fields of developmental, cellular, and molecular biology. Yet, despite decades of research, only recent attention has been given to its ecology, natural diversity, and evolutionary history (Frézal & Félix, 2015; Petersen et al., 2015; Schulenburg & Félix, 2017). Early surveys of wild *C*. *elegans* were largely focused on sampling from compost heaps in rural gardens. More recently, natural substrates in less artificial habitats have been surveyed revealing that wild *C*. *elegans* are typically found on rotting fruit and vegetable matter, where they persist by feeding on the diverse bacterial communities associated with these substrates (Schulenburg & Félix, 2017). However, intensive surveys of wild *C*. *elegans* in less artificial habitats have mostly been focused in Europe and the western continental United States, and relatively few strains have been isolated from other regions of the world (Barrière & Félix, 2005; Crombie et al., 2019; Félix & Duveau, 2012; Frézal & Félix, 2015; Kiontke et al., 2011; Petersen et al., 2014; Richaud et al., 2018; Sivasundar & Hey, 2005).

As the number of wild isolates has grown, analysis of the genetic diversity revealed a striking pattern of exceptionally low diversity in strains isolated from across much of the globe and higher diversity in strains isolated from the Hawaiian Islands and more generally across the Pacific Rim (Crombie et al., 2019; Lee et al., 2021). This pattern is thought to be explained at least in part by recent chromosome‐scale selective sweeps that have purged diversity from the species in many regions outside of the Pacific Rim and are speculated to have occured while the *C*. *elegans* range expanded in association with humans (Andersen et al., 2012). However, the evolutionary factors contributing to the exceptional genetic diversity on the Hawaiian Islands are not currently understood, but in general, elevated intraspecific genetic diversity is not uncommon to volcanic archipelagos (Shaw & Gillespie, 2016).

The Hawaiian Islands form the most remote volcanic archipelago in the world and the phylogeographic pattern that predominates for native Hawaiian taxa is that lineages tend to progress down the island chain, with the most ancestral groups (populations or species) on the oldest islands (Cowie & Holland, 2008; Roderick & Gillespie, 1998; Shaw & Gillespie, 2016). This progression of lineages is known as the “progression rule” and is characterized by genetic differentiation of taxa as successive islands are colonized (Funk & Wagner, 1995; Whittaker et al., 2008). On the Hawaiian Islands, natural selection is often evoked as a primary force of change during the initial differentiation of lineages (Roderick & Gillespie, 1998), but neutral forces such as repeated founder events and genetic drift may also contribute. Importantly, for any one taxa, patterns of diversity consistent with the progression rule can be obscured by more recent gene flow between islands or from the mainland. Even in the face of gene flow, elevated archipelago‐wide genetic diversity can persist, especially when diverse niches with strongly heterogeneous selection pressures are present.

A recent survey of *C*. *elegans* genetic diversity on the Hawaiian Islands showed that the genetic structure did not strictly conform to the expectations of the progression rule. That is, the patterns of differentiation did not associate with island age, as three of the four genetically distinct groups were found distributed across the islands (Crombie et al., 2019). Instead, the authors observed weak correlations between environmental variables at sampling locations and the genetic groups to which individuals were assigned. Although that analysis was based on a relatively small sample size, the results suggest that *C*. *elegans* deviates from the typical progression patterns observed for many taxa on the Hawaiian Islands and that local adaptation to heterogeneous environments could be an important driver underlying the exceptional genetic diversity observed among these strains.

Local adaptation can occur in response to varying selection pressures imposed by spatially heterogeneous environments and can cause alleles to vary in frequency across the range of a species (Kawecki & Ebert, 2004). For this reason, alleles correlated with features of the environment are often interpreted as a signature of local adaptation (Booker et al., 2021; Coop et al., 2010). However, disentangling the signatures of local adaptation from patterns caused by neutral forces and/or demographic histories can be difficult (Rellstab et al., 2015). To address this challenge, various genotype‐environment association (GEA) methods have been developed to detect genomic signatures of local adaptation and account for the effects of other forces where possible (Hoban et al., 2016; Rellstab et al., 2015). In recent years, these tools have been applied to help detect the genetic basis and modes of adaptation to environmental variation for several species that are important to the ecology, evolution, and conservation biology fields (Hancock et al., 2011; Rellstab et al., 2015).

To better characterize the exceptional patterns of genetic diversity on the Hawaiian Islands and to more thoroughly explore the role of local adaptation in shaping this diversity, we repeatedly sampled nematodes over a three‐year period, measured various environmental niche parameters at each sampling site, and whole‐genome sequenced *C*. *elegans* isolates. We found that *C*. *elegans* environmental niche preferences differ substantially from the other selfing *Caenorhabditis* species (*C*. *briggsae* and *C*. *tropicalis*) on the Hawaiian Islands. Surprisingly, *C*. *elegans* were enriched in native habitats and the other two species were enriched in introduced and disturbed habitats. We also observed considerable environmental variation within the *C*. *elegans* niche itself. We measured genetic diversity and differentiation using whole‐genome sequences from 464 Hawaiian *C*. *elegans* strains we collected and 36 Hawaiian strains that were collected by our collaborators. We found that this sample of Hawaiian strains comprises 163 non‐redundant genome‐wide haplotypes, which we refer to as isotypes, representing an almost four‐fold increase in sample‐size relative to the most recent study of Hawaiian diversity (Crombie et al., 2019). Using principal components analysis (PCA), we found evidence of seven genetically distinct groups within the sample. Additionally, we found that the patterns of diversity on the Islands partially conform to expectations of the progression rule. Moreover, we identified overlapping regions of the genome that display signatures of local adaptation to various environmental variables, including elevation, temperature, and precipitation, using two GEA methods. Taken together, these results contribute to our understanding of the various evolutionary forces shaping genetic diversity on the Hawaiian Islands and provide clues about the functional genetic variation relevant to local adaptation.

## 2 MATERIALS AND METHODS

### 2.1 Strains

Isolated nematodes were grown at 20°C using the *Escherichia coli* OP50 bacterial strain spotted on modified nematode growth medium (NGMA), containing 1% agar and 0.7% agarose to prevent animals from burrowing (Andersen et al., 2014). All cryopreserved strains (Supporting Information 1) used in this study are available from the *C*. *elegans* Natural Diversity Resource (Cook et al.,^2017^).

### 2.2 Sampling strategy

We sampled Hawaiian nematodes on six occasions from August 2017 to January 2020. These sampling projects varied in size, with four larger projects, comprising more than 500 samples in August 2017, October 2018, October 2019, and December 2019, and two smaller projects, comprising fewer than 100 samples in August 2019 and January 2020. For each project, we chose sampling locations based on accessibility to hiking trails and by proximity to where *Caenorhabditis* nematodes had been collected previously (Andersen et al., 2012; Cook et al., 2016; Crombie et al., 2019; Hahnel et al., 2018; Hodgkin & Doniach, 1997). In the four larger projects, we attempted to sample broadly across different habitats, which in Hawaii tend to vary along gradients of elevation and exposure to trade winds. At each sampling location, we opportunistically sampled substrates known to harbour *Caenorhabditis* nematodes, including rotting fruits, seeds, nuts, flowers, stems, mixed vegetal litter, compost, wood, soil, fungus, live arthropods, and molluscs (Crombie et al., 2019; Ferrari et al., 2017; Schulenburg & Félix, 2017). The mixed vegetal litter category describes substrates that contain detritus or dead organic material that forms a layer over the soil at most collection sites.

### 2.3 Field sampling and environmental data collection

We collected samples from nature by transferring substrate material directly into a pre‐barcoded collection bag as described previously (Crombie et al., 2019). When collecting substrates, we attempted to collect at least a tablespoon of material but did not weigh the material in the field. To characterize the abiotic niche of *Caenorhabditis* nematodes, we collected data for environmental parameters at each sampling site, including the surface temperature of the sample using an infrared thermometer (Lasergrip 1080, Etekcity) and the ambient temperature and humidity near the sample using a combined thermometer and hygrometer device (GM1362, GoerTek). We used a mobile device and a geographical data collection application, Fulcrum, to record the environmental parameter values, substrate GPS coordinates, *in situ* photographs of the substrate, and categorical descriptions of the substrate in a cloud database. We then exported the collection data from the Fulcrum database and processed it using the easyFulcrum (v1.0.0) R package to flag and correct anomalous data records (Di Bernardo et al., 2021). For *C*. *elegans* positive samples, we used a hierarchical clustering approach to group samples within a 3 km distance. We used the *distm()* function from the geosphere (v1.5–10) r package to calculate a geodesic distance matrix from the sample locations and then clustered the samples within 3 km groups using the *hclust()* and *cutree()* functions from the stats (v3.6.3) package. We chose to cluster with the 3 km distance because it reduced the within cluster sum of squares when samples were grouped by island and largely recapitulated the distinct hiking trail or region where the samples were collected.

### 2.4 GIS environmental data

To further characterize the environmental conditions near each sampling site, we used publicly available geographic information system (GIS) data and processed these data in r (v3.6.3) with the raster (v3.1‐5) and sf (v0.9‐5) packages (Hijmans, 2020; Pebesma, 2018; R Core Team, 2020). We used GIS maps produced for the assessment of evapotranspiration in the state of Hawaii at 250 m resolution to assess various average annual climate parameter values for our sampling sites, including air temperature, surface temperature, available soil moisture, and leaf area index (LAI; Giambelluca et al., 2014). LAI quantifies the amount of vegetation in a given area as the ratio of one‐sided leaf area to ground area. We also used GIS data from the Rainfall Atlas of Hawaii to determine mean annual rainfall totals at approximately 250 m resolution (Frazier et al., 2016; Giambelluca et al., 2013) and two GIS maps generated for the Carbon Assessment of Hawaii (CAH) to determine the land cover and habitat status at our sampling sites at 30 m resolution (Jacobi et al., 2017). We use the term land cover to describe characteristics of the plant communities within each 30 m map unit and the term habitat status to describe the condition of those plant communities. The CAH land cover map uses a hierarchical classification system that allows the user to group the mapped units into different configurations, including 48 detailed plant community units, 27 generalized land cover units, 13 biome units, and seven major land cover units. The CAH habitat status map depicts the distribution of plant communities that are (1) dominated by native species, (2) mixed native and alien species, (3) heavily disturbed areas with few native species, and (4) areas with less than five percent vegetation cover. We renamed classifications from the CAH habitat status map to native, introduced, disturbed, and bare. Bare habitats contain little vegetation mostly due to recent lava flows, and we did not include this habitat class in enrichment analyses because it was sampled infrequently (10 samples) and *Caenorhabditis* nematodes were never found there.

### 2.5 Nematode isolation

Following sample collection, bagged and barcoded samples were shipped overnight from Hawaii to Northwestern University where the substrates were transferred from the barcoded collection bags to matching barcoded 10 cm NGMA plates (Andersen et al., 2014) seeded with OP50 bacteria. We transferred one tablespoon of substrate material to the 10 cm NGMA collection plates. However, the volume of material collected for rotting flowers was often less than a tablespoon. Therefore, it is possible that these differences in material volume could bias our detection of nematodes in flower substrates relative to other substrate classes. We attempted to isolate nematodes from collection plates two days after the substrates were transferred. If no nematodes were found, we attempted to isolate nematodes again after seven days. For each collection plate, up to seven gravid nematodes were isolated by transferring them individually to prebarcoded 3.5 cm NGMA isolation plates seeded with OP50 bacteria. In many cases, gravid adult animals were not found on the collection plates, so we isolated larval stages instead. This technique biases our isolation strategy towards selfing nematode species. At the time of isolation, we scanned the barcodes on the collection and isolation plates with the Fulcrum mobile app so that each isolate was linked to the appropriate field collection record in the Fulcrum database. If we could not find nematodes on the collection plate after seven days, we recorded that the isolated nematode failed to proliferate. We exported the isolation data from the Fulcrum database and processed it with the easyFulcrum (v1.0.0) r package to join the isolation records with the collection records for further analysis (Di Bernardo et al., 2021).

### 2.6 Nematode identification

We identified *Caenorhabditis* isolates to the species‐level and isolates of other genera to the genus‐level by analysis of the internal transcribed spacer (ITS2) region between the 5.8S and 28S rDNA genes (Barrière & Félix, 2014; Kiontke et al., 2011). The isolated nematodes were stored at 20ºC for up to 21 days before they were genotyped but were not passaged during this time to avoid multiple generations of proliferation. For genotyping, we lysed three to five nematodes from an isolation plate in 8 µl of lysis solution (100 mM KCl, 20 mM Tris pH 8.2, 5 mM MgCl_2_, 0.9% IGEPAL, 0.9% Tween 20, 0.02% gelatin with proteinase K added to a final concentration of 0.4 mg/ml) then froze the solution at –80ºC for up to 12 h. If isolated nematodes could not be found on the isolation plates, we categorized them as “Not genotyped”. We loaded 2 µl of thawed lysis material into 40 µl reactions with primers spanning a portion of the ITS2 region using forward primer oECA1687 (CTGCGTTATTTACCACGAATTGCARAC) and reverse primer oECA202 (GCGGTATTTGCTACTACCAYYAMGATCTGC) (Kiontke et al., 2011). We also loaded 2 µl of the lysed material into 40 µl reactions with a second set of primers that amplify about 500 bp of 18S rDNA in Rhabditid nematodes using forward primer oECA1271 (TACAATGGAAGGCAGCAGGC) and reverse primer oECA1272 (CCTCTGACTTTCGTTCTTGATTAA) (Haber et al., 2005). The PCR conditions for both primer sets were described previously (Crombie et al., 2019). Products from both PCR amplifications were visualized on a 2% agarose gel in 1x TAE buffer. We classified isolates that did not produce bands with the either primer set as “unknown nematode” and isolates for which the 18S region amplified, but the ITS2 region did not, as “non‐*Caenorhabditis*”. The isolates that produced bands with both primer sets were investigated further using Sanger sequencing of the ITS2 PCR products with forward primer oECA306 (CACTTTCAAGCAACCCGAC). We compared these ITS2 sequences to the National Centre for Biotechnology Information (NCBI) database using the BLASTn algorithm, which identified *Caenorhabditis* isolates to the species‐level. Isolates with sequences that aligned best to genera other than *Caenorhabditis* were only identified to the genus‐level. In most cases, isolates identified as *C*. *briggsae* *,* *C*. *elegans*, or *C*. *tropicalis* were named and cryopreserved. For each named strain, one of four recently starved 10 cm NGMA plates was used to cryopreserve the strain, and the other three plates were used for DNA extraction and whole‐genome sequencing. Each strain underwent at least one population bottleneck and between three to five generations prior to cryopreservation and genomic DNA extraction.

### 2.7 Illumina library construction and whole‐genome sequencing

To extract DNA, we transferred nematodes from three recently starved 10 cm NGMA plates originally spotted with OP50 *E*. *coli* into a 15 ml conical tube by washing with 10 ml of M9. We then used gravity to settle animals in the conical tube, removed the supernatant, and added 10 ml of fresh M9. We repeated this wash method three times to serially dilute the *E*. *coli* in the M9 and allow the animals time to purge ingested *E*. *coli*. Genomic DNA was isolated from 100 to 300 µl nematode pellets using the Blood and Tissue DNA isolation kit (cat no. 69506, Qiagen) following established protocols (Cook et al., 2016). The DNA concentration was determined for each sample using the Qubit dsDNA Broad Range Assay Kit (cat no. Q32850, Invitrogen). Sequencing libraries were either generated with KAPA Hyper Prep kits (Kapa Biosystems), Illumina Nextera Sample Prep Kit (Illumina, Inc.), or New England BioLabs NEBNext Ultra II FS DNA Library Prep (NEB). Samples were sequenced at the Duke Center for Genomic and Computational Biology, Novogene, or the Northwestern Sequencing facility, NUSeq. All samples were sequenced on the Illumina HiSeq 4000 or NovaSeq 6000 platform (paired‐end 150 bp reads). The raw sequencing reads for strains used in this project are available from the NCBI Sequence Read Archive (Project PRJNA549503).

### 2.8 Variant calling

To ensure reproducible data analysis, all genomic analyses were performed using pipelines generated in the Nextflow workflow management system framework (Di Tommaso et al., 2017). Full descriptions of the pipelines can be found on the Andersen laboratory dry guide (http://andersenlab.org/dry‐guide/latest/pipeline‐overview/). Raw sequencing reads were trimmed using fastp, which removed low‐quality bases and adapter sequences with the trim‐fq‐nf pipeline. The trimmed reads were mapped with bwa (v0.7.17) (Li, 2013) to the N2 reference genome (WS276) with the alignment‐nf pipeline. Next, we used the wi‐gatk pipeline to call single nucleotide variants (SNVs) with GATK4 (v4.1.4) software following previously described methods (Lee et al., 2021). Because outcrossing rates in *C*. *elegans* are low, we expect the vast majority of the sites to be homozygous (Richaud et al., 2018). When heterozygous sites are detected in the raw read alignments, we use the log‐likelihood ratios of reference to alternative genotype calls to convert these sites to be homozygous for the most likely allele (Cook et al., 2016). When the log‐likelihood ratio was <−2 or >2, heterozygous genotypes were converted to reference genotypes or alternative genotypes, respectively. All other SNVs with likelihood ratios between −2 and 2 were left as heterozygous variants. Very few sites remain heterozygous after this correction. Further details can be found on the CeNDR website (https://www.elegansvariation.org). After variant calling, the following filters were applied with GATK4 to keep only high‐quality variants: read depth (FORMAT/DP >5); variant quality (INFO/QUAL >30 and quality by depth INFO/QD >20); and strand bias (INFO/FS <100 and INFO/SOR <5). Variant sites that have a missing genotype in more than 95% of samples or are heterozygous in more than 10% of samples were also removed. With the high‐quality set of variants, we ran the concordance‐nf pipeline to compare *C*. *elegans* strains isolated in this study and previously described strains (Cook et al., 2016, 2017; Hahnel et al., 2018; Lee et al., 2021). We classified two or more strains as the same isotype if they shared >99.97% of SNVs. If a strain did not meet this criterion, we classified it a unique isotype. All genetic analyses in this paper were done on the isotype level. We refer to the final isotype‐level VCF as the “isotype VCF”.

### 2.9 Tree‐based analyses

We characterized the relatedness of the *C*. *elegans* isotypes using quicktree (v2.5) software (Howe et al., 2002). To construct the unrooted tree that includes 540 isotypes (Figure S11), we used SNVs from the “isotype VCF” that were converted to the phylipformat (Felsenstein, 1993) using the vcf2phylip.py script (Ortiz, 2019). This tree was visualized using the ggtree (v1.10.5) r package (Yu et al., 2017). To construct the unrooted trees of 163 Hawaiian isotypes (Figure S13), we further pruned the “isotype VCF” by filtering to biallelic SNVs only and removing sites in linkage disequilibrium (LD) using the plink (v1.9) commands (‐‐snps‐only ‐‐biallelic‐only ‐‐indep‐pairwise 50 10 0.8). This ‐‐indep‐pairwise command uses 50 marker windows and greedily prunes variants from this window that have *r* ^2^ values greater than the threshold (0.8) until no such pairs remain. Then, the sliding window steps forward 10 markers and repeats the process. We also used various pairwise *r* ^2^ thresholds (0.8, 0.6, 0.2, and 0.1) to explore the effects of LD pruning on tree topology (Figure S13).

### 2.10 Population genetic statistics

Genome‐wide nucleotide diversity (∏) and Tajima's *D* were calculated using the vcftools (v0.1.15) software. We calculated these statistics separately for the 163 Hawaiian isotypes and the 377 non‐Hawaiian isotypes by subsetting the full “isotype VCF”. We then calculated genome‐wide ∏ along sliding windows with a 10 kb window size and a 1 kb step size using the (‐‐window‐pi 10000 ‐‐window‐pi‐step 1000) commands for both groups. We also calculated Tajima's *D* with a 10 kb window size without sliding using the (‐‐TajimaD 10000) command for both groups. All statistics were computed for all sites regardless of annotation status, *i*.*e*., we did not consider intronic, non‐coding, RNA gene, synonymous, or nonsynonymous sites separately.

### 2.11 Principal components analysis and population structure

The smartpca executable from eigensoft (v6.1.4) was used to perform PCA (Price et al., 2006). We performed this analysis with the “isotype VCF” that was subset to the 163 Hawaiian isotypes then filtered to biallelic snps only using the plink (v1.9) commands (‐‐snps‐only ‐‐biallelic‐only) and LD pruned with the command (‐‐indep‐pairwise 50 10 0.1). We ran smartpca with and without removing outlier isotypes to analyse the population structure among the Hawaiian isotypes. When analysing the population without removing outlier isotypes, we used the following parameters: altnormstyle: NO, numoutevec: 50, familynames: NO, numoutlieriter: 0. When analysing the population with outlier isotype removal, we set numoutlieriter to 15. We performed hierarchical cluster analysis on the significant eigenvectors in r using the stats package hclust function with the “average” agglomeration method and cut the tree with the cutree function and “k = 7” (R Core Team, 2020). Isotypes were assigned to genetic groups based on the clusters.

### 2.12 Genotype‐environment association and local adaptation

We used two GEA methods (BayPass and GWA) to scan the genome for signatures of local adaptation. Prior to performing GEA with BayPass and GWA, we further pruned the “isotype VCF” by filtering to biallelic SNVs only, removing sites in LD, and setting a minor allele frequency cutoff using the plink (v1.9) commands (‐‐snps‐only ‐‐biallelic‐only ‐‐indep‐pairwise 50 1 0.8 ‐‐maf 0.1). We used baypass (v2.2) (Gautier, 2015) to perform GEA with SNV data from isotypes that were found in 3 km sampling clusters that contained at least three isotypes. This filtering strategy resulted in 142 isotypes from 13 sampling clusters for use in BayPass. To generate a scaled population covariate matrix (Ω matrix), we first ran BayPass with the core model using 13 populations (3 km sampling clusters) and a subsampled data set of 5,000 biallelic SNVs from outside annotated gene coding regions generated using the plink (v1.9) commands ‐‐exclude and ‐‐thin‐count 5000 The Ω matrix was then used to explicitly account for the covariance structure in population allele frequencies resulting from the demographic history of the populations in subsequent BayPass runs. We reran the BayPass core model using the full set of biallelic SNVs, 13 populations, and the Ω matrix. The BayPass core model outputs XtX statistics for each marker that can be used to identify differentiation caused by selection rather than other processes. We considered XtX statistic values as suggestive of local adaptation if they were among the top 0.1% of genome‐wide XtX statistic values. We then ran BayPass again using the standard covariate model with biallelic SNV data, 13 populations, the Ω matrix, and eight environmental variables as covariates (altitude, mean annual air temperature, mean annual surface temperature, mean annual rainfall, mean annual soil moisture, mean annual leaf area index, latitude, and longitude). We considered Bayes factors (BFs) above 20 as evidence of a significant genotype‐environment association. We also performed GEA with the NemaScan pipeline, a gwa tool specifically designed for *C*. *elegans*, which is available at: https://github.com/AndersenLab/NemaScan (Widmayer et al., 2021). NemaScan uses both the ‐‐mlma‐loco and ‐‐fastGWA‐lmm‐exact functions from gcta (v1.92.3beta2) software to perform rapid GWA (Yang et al., 2011). The ‐‐mlma‐loco function accepts a limited sparse kinship matrix composed of all chromosomes except the chromosome containing the tested marker (LOCO = “leave one chromosome out”) and the ‐‐fastGWA‐lmm‐exact accepts a full sparse kinship matrix specifically calculated for inbred model organisms. These functions can account for population structure in the mapping sample but use a different strategy than BayPass. To determine significant markers, we used eigen decomposition of the kinship matrix to correct for the number of independent tests in each mapping, as was described previously (Zdraljevic et al., 2019).

To identify genomic regions of interest that contained markers significantly associated with environmental parameters, we grouped markers into a single region if they were within 1 kb of each other and were above the significance threshold. We then expanded that region of interest to include the portions of the genome containing 150 markers to the left and right of its original range. Ultimately, we identified overlapping regions of interest for the two GEA methods using the bedtools (v2.30) intersect command.

## 3 RESULTS

### 3.1 Hawaiian nematode diversity

**FIGURE 1:**
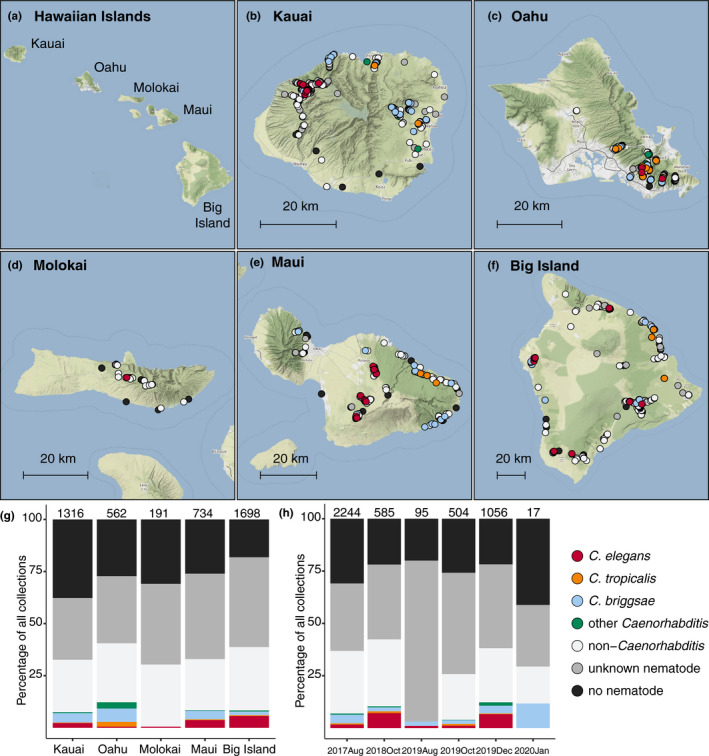
Geographic and temporal distribution of sampling sites across the Hawaiian Islands. (a) An overview of the Hawaiian Islands. (b–f) Detailed views of each of the islands sampled. Circles indicate sampling sites and are coloured according to the legend below. We categorized nematodes as “other *Caenorhabditis*” if they did not belong to one of the three selfing *Caenorhabditis* species and “non‐*Caenorhabditis*” if their ITS2 region aligned to genera other than *Caenorhabditis* or if the ITS2 region failed to amplify but the 18S region did. We categorized nematodes as “unknown nematodes” if we could not extract high‐quality genomic DNA or amplify either region by PCR (see Materials and Methods). For sampling sites where multiple collection categories apply (*n* = 733), the site is coloured by the collection category shown in the legend from top to bottom, respectively. (g–h) The percentage of each collection category is shown by island (g) or collection project (h). Bars are coloured according to the legend on the right and the total number of samples for each category are shown above the bar

We collected Hawaiian nematodes on six occasions between August 2017 and January 2020. On each collection trip, we performed multiple sampling trials in different habitats and preferentially sampled substrates known to harbour *Caenorhabditis* nematodes, including rotting fruits, seeds, nuts, flowers, stems, mixed vegetal litter, compost, wood, soil, fungus, live arthropods, and molluscs (Crombie et al., 2019; Ferrari et al., 2017; Schulenburg & Félix, 2017). At each sampling site, we measured various environmental parameters, including ambient humidity and temperature, elevation, and substrate temperature using hand‐held devices (Di Bernardo et al., 2021). We sampled from some hiking trails on multiple collection trips, but other trails were only sampled once. Overall, we sampled 4506 substrates across five Hawaiian Islands (Figure 1) and isolated 7107 nematodes from 2400 of these substrates (53.3% success rate for nematode isolation). We attempted to identify these isolates by analysis of the 18S rDNA gene and the internal transcribed spacer (ITS2) region between the 5.8S and 28S rDNA genes (see Materials and Methods) (Barrière & Félix, 2014; Kiontke et al., 2011). We identified *Caenorhabditis* isolates to the species‐level and isolates from other genera to the genus‐level. In total, we identified 23 distinct taxa across the Hawaiian Islands, including five *Caenorhabditis* species that were found at different frequencies among the 4506 substrates sampled: *C*. *briggsae* (3.6%), *C*. *elegans* (3.6%), *C*. *oiwi* (0.68%), *C*. *tropicalis* (0.58%), and *C*. *kamaaina* 0.09%) (Table S1). We also collected 13 samples (0.29%) harbouring 31 isolates with ITS2 regions that were most closely related to either *C*. *plicata* or *C*. *parvicauda* but at such low identity that we suspect the isolates belong to one or more new *Caenorhabditis* species. However, all of these isolates perished before they could be cryopreserved so we have classified them as *“Caenorhabditis* spp.”.

Our collection data suggest that nematode diversity is similar across the five sampled islands. The number of taxa identified on each island scaled with the number of samples collected and ranged from 20 taxa on the Big Island to just three taxa on Molokai. However, we did not detect an enrichment of diversity on any particular island when considering the number of taxa relative to the number of genotyped isolates from the island (Fisher's exact test; *p* > .05 for all island comparisons). We also looked for differences in diversity at a finer geographic scale (~3 km) by comparing diversity among various sampled trails. We isolated between zero and 11 taxa per trail, but we found no evidence of enrichment on any particular trail (Fisher's exact test; *p* > .05 for all trail comparisons). In rare cases, we observed nematode diversity at the substrate scale (~10 cm). Among the 4506 substrates sampled, we observed 46 instances of distinct taxa cohabitating on the same substrate, a finding consistent with previous surveys of *Caenorhabditis* nematodes (Crombie et al., 2019; Félix et al., 2013; Petersen et al., 2014; Richaud et al., 2018). Among these samples, 21 contained more than one *Caenorhabditis* species (Figure S1). These patterns of *Caenorhabditis* nematode diversity suggest that species within the genus could compete for resources across multiple spatial scales. Given the high frequencies of colocalization among *Caenorhabditis* species, we searched for observable differences in environmental niche preferences among them.

### 3.2 *Caenorhabditis* niche specificity

**FIGURE 2:**
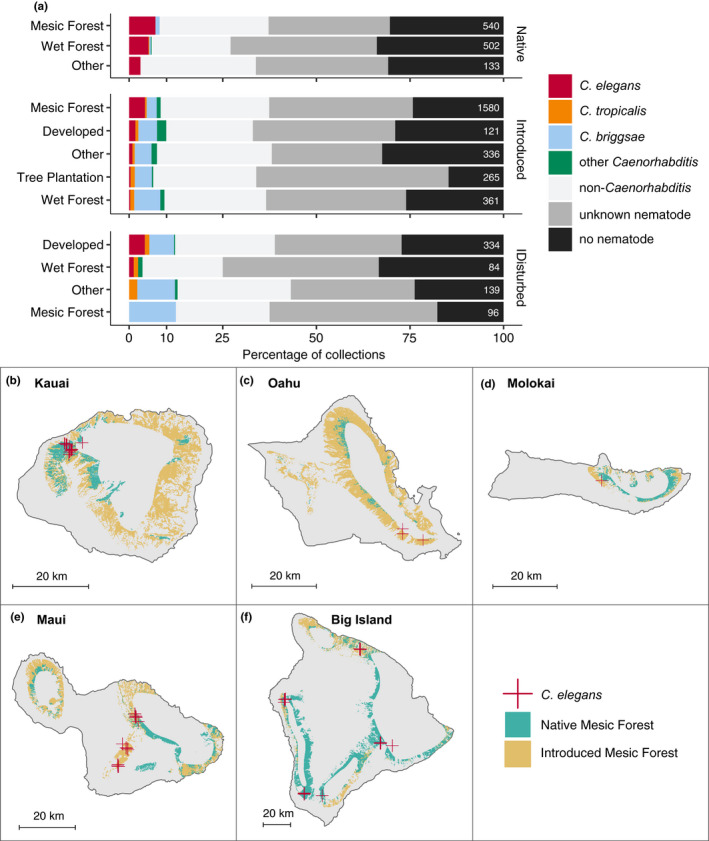
Land cover enrichment among selfing *Caenorhabditis* nematodes. (a) The percentage of each sampling category is shown by land cover type. The land cover types are organized by native, introduced, and disturbed habitats. The sampling categories are coloured according to the legend at the right, and the total number of samples for each substrate are shown on the right side of the bars. (b–f) Land cover maps for each of the five Hawaiian Islands sampled in this study. The sample locations where *Caenorhabditis elegans* were found are shown as red crosses, and native and introduced mesic forest land covers are shaded blue and orange, respectively

To characterize the niche preferences of *Caenorhabditis* nematodes, we used publicly available geospatial data to identify habitats where they are found most frequently. We used a GIS map of habitat status to assign each sampling site one of three habitat conditions: native, introduced, or disturbed (see Materials and Methods) (Jacobi et al., 2017). Native habitats are dominated by native Hawaiian plant communities; introduced habitats contain a mixture of native and introduced species; and disturbed habitats are impacted by agriculture or urban development. Overall, we found *Caenorhabditis* nematodes were enriched in disturbed habitats (74 of 653 11.3%) relative to native habitats (78 of 1175 6.6%) but not relative to introduced habitats (221 of 2663 8.3%) (Fisher's exact test; disturbed vs native *p* = .0044, disturbed vs. introduced *p* =  0.10). The pattern of habitat enrichment was strikingly different for *C*. *elegans* relative to the other selfing species. Whereas *C*. *briggsae* and *C*. *tropicalis* were enriched in disturbed habitats relative to native habitats, *C*. *elegans* was enriched in native habitats (68 of 1175 5.8%) relative to introduced (74 of 2263 2.8%) or disturbed habitats (15 of 653 2.3%) (Fisher's exact test, *p* < .05 for all comparisons) (Figure 2a, Figure S2). We also used land cover GIS data to determine whether *Caenorhabditis* nematodes were associated with a particular land cover class (Jacobi et al., 2017). We found no evidence of *Caenorhabditis* enrichment for any particular land cover within native, introduced, or disturbed habitat classes. However, *C*. *elegans* were found most frequently in mesic forests compared to the other land covers within the native and introduced habitats. Hawaiian mesic forests have moderate amounts of rainfall (1200–2500 mm annually) and are found on leeward and windward sides of the islands in lowland or in montane‐subalpine zones (Cuddihy et al., 1990). Among introduced habitats, *C*. *elegans* were significantly enriched in mesic forests relative to all other land covers except developed (Fisher's exact test; *p* < .05 for significant comparisons) (Figure 2a). Notably, the number of samples we collected from introduced habitats with developed land cover was small (*n* = 121) and both samples that contained *C*. *elegans* (*n* = 2) were taken from the roadside adjacent to mesic forest land cover. Taken together, these data suggest that *C*. *elegans* niche preferences might be different from other selfing *Caenorhabditis* species on the Hawaiian Islands. *C*. *elegans* seems to prefer native mesic forest habitats, but *C*. *briggsae* and *C*. *tropicalis* are more frequently found in disturbed and introduced habitats and at similar frequencies across land covers within these habitats (Figure 2a).

**FIGURE 3:**
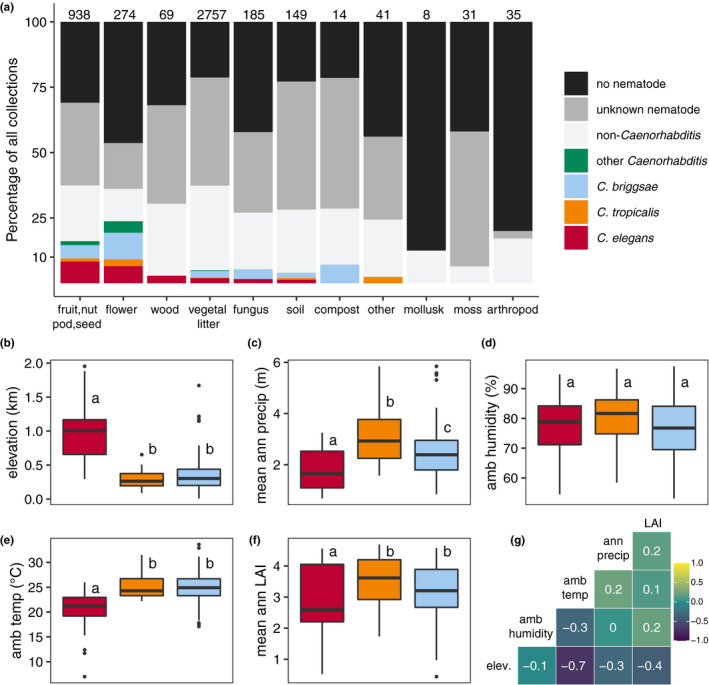
Niche differentiation among selfing *Caenorhabditis* nematodes. (a) The percentage of each sampling category is shown by substrate type. The sampling categories are coloured according to the legend on the right, and the total number of samples for each substrate are shown above the bars. (b–f) Environmental parameter values; elevation, mean annual precipitation, in situ ambient humidity, in situ ambient temperature, and mean annual leaf area index (LAI) for sites where *Caenorhabditis* species were isolated. Tukey's box plots are plotted by species (red is *Caenorhabditis elegans*, orange is *C*. *tropicalis*, blue is *C*. *briggsae*) for each environmental parameter; points above or below whiskers indicate outliers. Letters above the boxes summarize the statistical significance of comparisons between the species shown. Species with a different letter are significantly different; species with the same letter are not significantly different. Comparisons were made using a Kruskal‐Wallis test and Dunn's test for multiple comparisons with *p*‐values adjusted using the Bonferroni method. (g) A correlation matrix for the continuous environmental parameters shown. The parameter labels for the matrix are printed on the diagonal, and Pearson's correlation coefficients are printed in the cells. The colour scale also indicates the strength and sign of the correlations shown in the matrix

On the Hawaiian Islands, elevation and tradewinds influence species assemblages and land covers by causing gradients in various environmental parameters, including temperature, and rainfall (Lowe et al., 2020). To further characterize Hawaiian *Caenorhabditis* niche preferences, we measured various environmental parameters at each sampling site in situ, including elevation, substrate temperature, ambient temperature, and ambient humidity. We also obtained measurements of additional continuous environmental parameters from geospatial models by extracting data associated with the location of each sampling site (see Materials and Methods, Figures S3 and S4; Frazier et al., 2016; Giambelluca et al., 2013, 2014). To reduce the dimensionality of these data, we choose to focus on just five of the nine variables by removing the variable with the most missing data and correlation with another variable was >0.7 (Figure 3b–g). We observed that on average, *C*. *elegans* were found at cooler, higher elevations in less densely vegetated and drier regions than the other selfing *Caenorhabditis* species (Kruskal‐Wallis and Dunn's post‐hoc test *p* < .05; ambient temperature, elevation, mean annual leaf area index, ambient humidity, mean annual precipitation). We sampled *C*. *elegans* from surprisingly cold substrates; three high‐elevation *C*. *elegans*‐positive collections from Maui (elevation >1400 m) made in December 2019 were temperature outliers among all selfing species. One of these substrates was collected at an ambient temperature of 7.0°C, with a substrate temperature of 3.9°C. We did not observe differences between *C*. *briggsae* and *C*. *tropicalis* species for the majority of environmental parameters, although all three species were differentiated from each other with respect to mean annual precipitation (Figure 3c). On average, *C*. *elegans* was found at sites with the lowest precipitation values relative to *C*. *briggsae* and *C*. *tropicalis* (Kruskal‐Wallis and Dunn's post‐hoc test, *p* < .05 for all comparisons). These trends suggest that wild *C*. *elegans* tend to prefer high altitude, cooler, native‐dominated, mesic forest habitats. *C*. *briggsae* and *C*. *tropicalis* are typically found at lower elevations in warmer, wetter, less native habitats. Consistent with these patterns, the cohabitation frequency between *C*. *tropicalis* and *C*. *briggsae* (2.6%, 5 of 190 collections) is higher than the cohabitation frequency between *C*. *elegans* and *C*. *briggsae* (0.9%, 3 of 326 collections) or *C*. *elegans* and *C*. *tropicalis* (0%, 0 of 188 collections). Moreover, the three samples harbouring both *C*. *elegans* and *C*. *briggsae* were collected between 650 and 800 m, near the lower range of *C*. *elegans* elevations and the upper range of *C*. *briggsae* elevations.

We also classified substrate types at each sampling location to explore possible preferences among the three selfing *Caenorhabditis* species. We isolated *Caenorhabditis* nematodes at higher frequencies from flower (65 of 274 or 23.7%) and fruit substrates (151 of 938 or 16.0%) than any other category except compost (Fisher's exact test, *p* < 0.05) (Figure 3a). Notably, the sample size for wood, compost, and other substrates was low, which limits our power to detect *Caenorhabditis* preferences for these substrates. These trends underscored known preferences for decomposing flower and fruit substrates identified by previous surveys of wild *Caenorhabditis* nematodes in tropical regions (Crombie et al., 2019; Félix et al., 2013; Ferrari et al., 2017). Although *Caenorhabditis* nematodes are associated with invertebrates in the wild (Kiontke & Sudhaus, 2006; Schulenburg & Félix, 2017), we did not isolate them from either molluscs (*n* = 8) or arthropods (*n* = 35), but our sample size of these substrates was also small (Figure 3a). Moreover, we did not explore whether viable nematodes were present internally within molluscs as has been documented for *C*. *elegans* and *C*. *briggsae* in Africa and Europe (Félix & Duveau, 2012; Petersen, Hermann, et al., 2015; Ross et al., 2012). We did not observe differences in the patterns of substrate enrichment among the selfing species; all species were found more frequently on fruit and flower substrates than vegetal litter (Fisher's exact test, *p* < .05; Figure 3a). This observation differs from our initial survey of Hawaiian nematodes, where we did not identify a significant substrate enrichment for *C*. *elegans* (Crombie et al., 2019). Importantly, the *C*. *elegans* enrichment in native habitats described above is not caused by oversampling of fruit or flower substrates in native habitats. In fact, we sampled fruit and flower substrates less frequently in native habitats than introduced or disturbed habitats (Figure S5). However, it is possible that unequal sampling of preferred substrates among different land covers could cause the enrichment of *C*. *elegans* in mesic forests, for example, we sampled the preferred fruit substrate class more frequently in native mesic forests than the other native land cover classes (Figure S6). Additional sampling of diverse substrate types across different land covers will be needed to address this possibility.

Overall, the sampling data reveal that *C*. *elegans* niche preferences are probably different from *C*. *briggsae* and *C*. *tropicalis* but also reveal considerable variation within the niche for each species. For example, although *C*. *elegans* are typically found in native habitats, a number of *C*. *elegans* were isolated from disturbed habitats in developed areas. Moreover, although *C*. *elegans* was on average found at cooler, higher elevations than other *Caenorhabditis* species, we isolated the species across a wide range of ambient air temperatures (7–26°C) and elevations (295–1950 m). We were curious whether the genetic diversity of Hawaiian *C*. *elegans* might be associated with variation in niche parameters on the islands. To explore this possibility, we sequenced the genomes of the *C*. *elegans* strains that we had isolated.

### 3.3 Genetic diversity of Hawaiian *Caenorhabditis elegans*

We sequenced the genomes of the 464 extant Hawaiian *C*. *elegans* strains to high coverage (median 27×) and identified single nucleotide variants (SNVs) and small indel variants in each genome relative to the N2 reference genome (36 strains were lost before cryopreservation). In *C*. *elegans*, some wild strain genome sequences are often nearly identical because of the high rates of self‐fertilization in the species. To reduce the number of invariant genomes in our analyses, we measured the concordance among all wild strain pairs and grouped strains sharing >99.97% of SNVs into a single genome‐wide haplotype that we refer to as an “isotype” (see Materials and Methods; Andersen et al., 2012). Using this strategy, we identified 143 Hawaiian isotypes among the 464 wild strains that we sequenced. We expanded the sample to 163 Hawaiian isotypes with 20 additional isotypes that were either sampled prior to 2017 or more recently by our collaborators. The vast majority of the Hawaiian isotypes comprise strains that were sampled from the same substrate (132 of 163, 82%). Among the 31 isotypes sampled from multiple substrates, 29 had location data for each substrate sampled. The median sampling distance between substrates within the same isotype was 35.9 m (Figure S7). Notably, only three isotypes comprised strains sampled more than 500 m from one another and each of these isotypes were sampled over multiple years. For example, isotype XZ1513 was first sampled in 2014 from the island of Kauai and then sampled again in 2018 from a location more than 6 km away from the original sampling location. Among the isotypes sampled from multiple substrates, the distribution of environmental parameters within each isotype was remarkably consistent (Table S2).

We applied hierarchical clustering methods to assign *C*. *elegans* positive samples to one of 21 discrete 3 km diameter sampling locations across the islands (see Materials and Methods). The vast majority of Hawaiian isotypes comprised strains that were sampled in close proximity to one another. Specifically, 155 of the 163 isotypes were never sampled from more than one of the 21 sampling locations, two isotypes were found in two sampling locations, and six isotypes could not be clustered because GPS positions were not available (Table S3, Figure S8). Similar to another longitudinal survey of genetic diversity in *C*. *elegans* (Richaud et al., 2018 *)*, we identified the same isotypes that persisted at sampling locations over multiple years (Figure S9). However, unlike previous studies, we never observed a single isotype that persisted at high frequency within a sampling location. In each of the five sampling locations where isotypes persisted, the average persistence time between the first and last isolation was 684 days, and in all cases, the relative abundance of the isotype in the sampling location varied substantially over time. We did not observe obvious seasonal variation in *C*. *elegans* abundance or relative abundance of isotypes at sampling locations, but these patterns might be observable with additional sampling at regular intervals. Overall, we found *C*. *elegans* isolation frequency increased from August to December when we pooled all sampling locations, but this finding is confounded by sampling bias towards preferred substrates in December (Figure S10). We found a high degree of genetic diversity within sampling locations relative to longitudinal surveys of diversity in Europe. Among the 10 sampling locations where we collected at least five samples, we found an average of 0.93 isotypes per sample. By comparison, the number of isotypes sampled from non‐Hawaiian sampling locations is often much lower with 0.29 isotypes per sample in France and 0.27 isotypes per sample in Germany (Andersen et al., 2012; Cook et al., 2016; Lee et al., 2021; Petersen et al., 2014; Richaud et al., 2018). The identification of more isotypes per sample on the Hawaiian Islands is not because we genotyped more individuals from a given sample. In fact, the number of individuals genotyped per sample was on average twice as high in the European locations (approximately five individuals per sample) as it was in our Hawaiian locations (approximately 2.5 individuals per sample). Taken together, these data suggest that genetic diversity at the local scale (1–10 km^2^) is higher on the Hawaiian Islands than it is in France and Germany.

Across a total of 163 Hawaiian isotypes, we identified 2.6 million SNVs and 507,680 small indels, which is twice the number of variants found among the 377 non‐Hawaiian isotypes known at the time of this study (Cook et al., 2017) (CeNDR release 20210121). Moreover, we found that approximately 60% of all SNVs and indels described in *C*. *elegans* are only found in Hawaiian isotypes and that nucleotide diversity (π) is almost three‐fold higher in the set of Hawaiian isotypes than it is among non‐Hawaiian isotypes (Hawaiian π = 0.0031; non‐Hawaiian π = 0.0012; Figure S11). Consistent with previous analyses of genetic diversity in *C*. *elegans*, we detected higher levels of diversity in both Hawaiian and non‐Hawaiian samples on chromosome arms relative to centers, a pattern that is probably caused by higher rates of recombination and therefore reduced levels of background selection on chromosome arms (Andersen et al., 2012; Crombie et al., 2019; Cutter & Payseur, 2003; Rockman et al., 2010). We also detected multiple localized spikes in π with values exceeding six times the genome‐wide average for both Hawaiian and non‐Hawaiian samples. These spikes in diversity often overlap with spikes in Tajima's *D* and probably reflect hyper‐divergent regions that are theorized to be maintained in *C*. *elegans* by long‐term balancing selection (Lee et al., 2021; Thompson et al., 2015). We suspect that the remarkable genetic diversity in the Hawaiian isotypes relative to the non‐Hawaiian isotypes is at least partially caused by the near absence of selective sweeps in the Hawaiian isotypes. These megabase‐scale selective sweeps are thought to have purged genetic diversity from many non‐Hawaiian isotypes across the centers of chromosomes I, IV, V, and the left arm of chromosome X (Andersen et al., 2012). The presence of these sweeps in the non‐Hawaiian isotypes is evident from the genomic pattern of Tajima's *D* where values are exceptionally low in the centers of chromosome I, IV, and V. In contrast, Tajima's *D* fluctuates near neutrality across these same chromosomal regions in the Hawaiian isotypes (Figure S12). Indeed, we found that 93% (351 of 377) of non‐Hawaiian isotypes have at least one swept chromosome compared to just 4% (6 of 163) of Hawaiian isotypes, and this pattern probably contributes to the high degree of differentiation for most Hawaiian isotypes (Figure S13). Consistent with the speculative theory that sweeps offer fitness advantages in human‐associated habitats (Zhang et al., 2021), four of the six isotypes with selective sweeps were isolated from disturbed or introduced habitats with developed land covers. Moreover, two of those isotypes (ECA923 and ECA928) were isolated from a backyard garden in downtown Honolulu. However, one isotype (XZ1515) with a global swept haplotype was isolated from a native mesic forest habitat in Kauai so globally swept haplotypes are also present in at least some native habitats on the Islands. Other studies have identified an excess of heterozygous loci in some *C*. *elegans* wild isolates and have used these data to estimate outcrossing rates in the wild, and have overwhelmingly found outcrossing rates to be low and effective recombination to be very low (Richaud et al., 2018). We did not observe any obvious signs of outcrossing within the genomes that we sequenced. However, we cannot be sure whether this result is caused by differences in our isolation strategy that could limit our ability to detect heterozygosity (see Materials and Methods). For this reason, we have not estimated outcrossing frequency with our Hawaiian isolates.

### 3.4 Genetic structure of Hawaiian *Caenorhabditis elegans*

**FIGURE 4:**
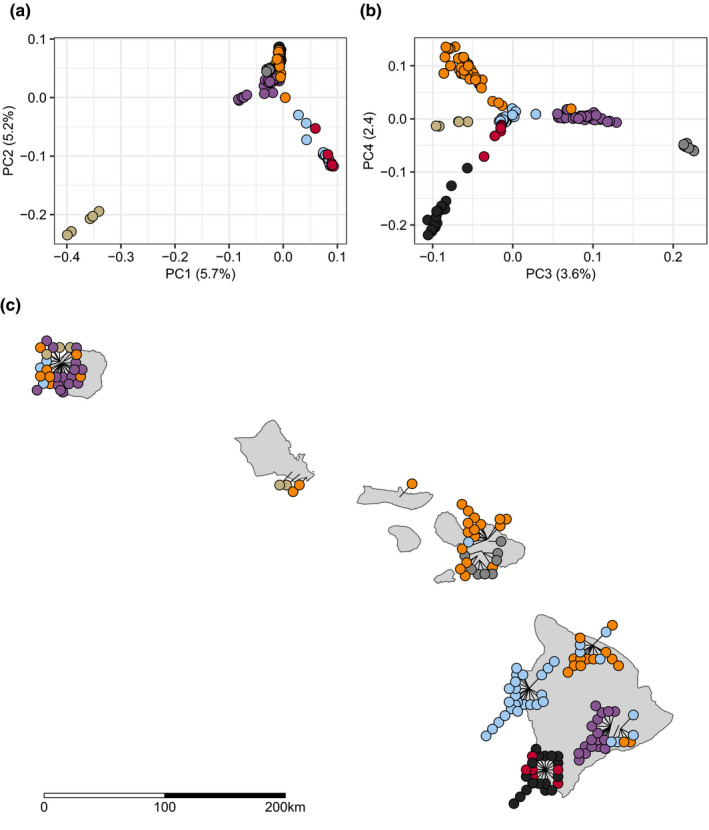
*Caenorhabditis elegans* genetic structure on the Hawaiian Islands. (a, b) Plots show major axes of variation derived from principal components analysis (PCA) of the genotype covariance matrix of 163 Hawaiian isotypes. (a) The first two axes of variation are plotted (PC1 and PC2). (b) The third and fourth axes of variation are plotted (PC3 and PC4). (a, b) The points indicate individual isotypes and are coloured by genetic group assignments obtained from hierarchical clustering of eigenvalues. Only 149 of 163 isotypes are shown, 14 outlier isotypes were removed from the PCA (see Materials and Methods). (c) The sampling locations for 144 of 163 Hawaiian isotypes are plotted on the Hawaiian Islands. Each circle represents a single isotype and is coloured by genetic group assignment. The 14 isotypes that are PCA outliers and five isotypes without location data are not shown

**FIGURE 5:**
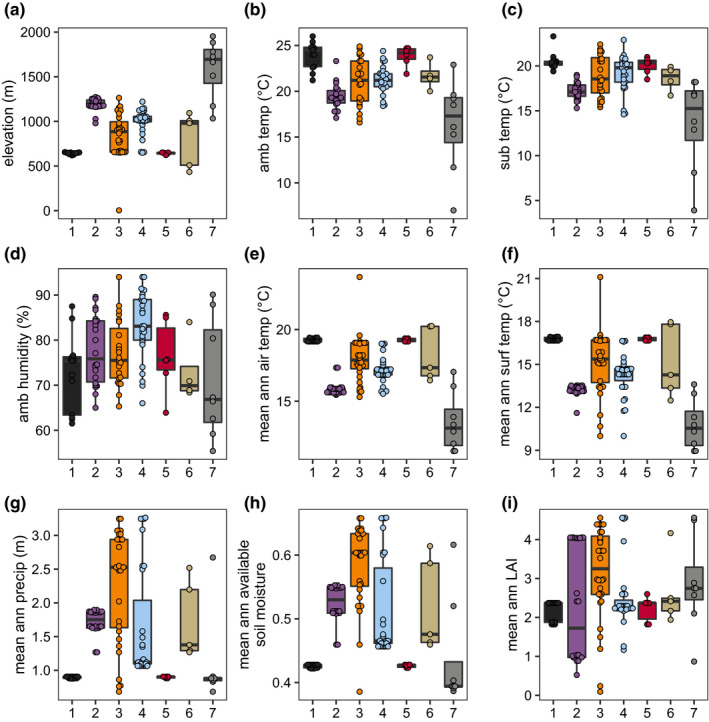
Environmental parameters for *Caenorhabditis elegans* genetic groups. Environmental parameter values measured at the time of collection: elevation (a), ambient temperature (b), substrate temperature (c), ambient humidity (d). Environmental parameter values obtained from environmental models; mean annual air temperature (e), mean annual surface temperature (f), mean annual precipitation (g), mean annual available soil moisture (h), mean annual leaf area index (i). Tukey's box plots are plotted by genetic group assignment from PCA (colours) for each environmental parameter

**FIGURE 6:**
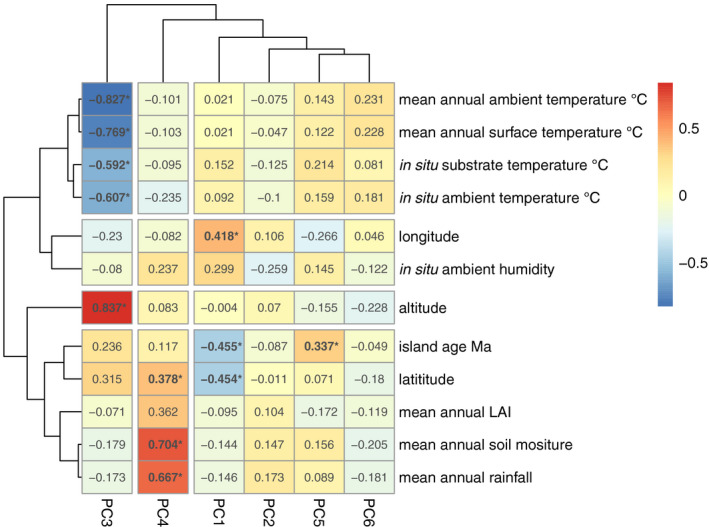
PC and environmental parameter correlation heatmap. The six significant axes of genetic variation (PCs) from PCA on 149 non‐outlier Hawaiian isotypes are shown on the x‐axis (PCs 1–6), and continuous environmental parameters are plotted on the y‐axis. The cell values represent the Pearson's correlation coefficient between a given PC and environmental parameter values, bold values with an asterisk indicate a significant correlation. The cell colours correspond to the strength and direction of the correlation (see colour scale on the right)

We examined the genetic structure among the 163 Hawaiian isotypes using PCA (Price et al., 2006). We found six significant principal components (PCs) that together explain 21% of the genetic variation within the Hawaiian isotypes. We then applied hierarchical clustering to these axes of variation and subdivided the Hawaiian sample into seven genetically distinct groups (Figure 4, Figures S14 and S15). To explore whether these genetic groups were associated with geography, we plotted isotype sampling coordinates onto a map of Hawaii and coloured them by their genetic group assignments (Figure 4c). We found that some genetic groups were widely distributed across the islands, and others were restricted to individual sampling locations. For example, the orange group consisted of 42 isotypes that were sampled from all five islands. By contrast, the red and black groups consisted of six and 17 isotypes, respectively, and each were sampled exclusively from Manuka State Wayside Park located on the south side of the Big Island. We examined the distributions of environmental parameter values for each of the genetic groups and found clear differences among groups, suggesting that the groups could be adapted to specific niches across the islands (Figure 5). We next tested whether the PCs were correlated with either spatial coordinates, continuous environmental parameters, or the age of the islands. We found that the major axis of genetic variation, PC1, explained 5.7% of the total genetic variation and was significantly correlated with age of the islands, longitude, and latitude (Figure 6). The correlations of PC1 with island age and geography are consistent with the expectations of the progression rule, lending support to the theory that *C*. *elegans* could be a native Hawaiian species. We also detected significant correlations between other axes of genetic variation and continuous environmental parameters. For example, PC3 is positively correlated with altitude and negatively correlated with various measures of temperature at isotype sampling sites. Moreover, PC4 is positively associated with measures of moisture at isotype sampling sites. Together, these data suggest that genetic variation among the Hawaiian isotypes could be partially explained by local adaptation to environmental conditions at sampling locations, although these correlations might also be caused by neutral processes. The purple group offers another compelling indication that local adaptation contributes to the genetic variation that we observe on the Hawaiian Islands. It comprises 34 isotypes that were sampled from two locations separated by over 500 km, the first on Kauai (15 isotypes) and the other on the Big Island (17 isotypes) (Figure 4). Despite the extreme distance separating these sampling locations, they have similar environments with respect to elevation, temperature, and moisture (Figure 5). Moreover, both locations contain high‐elevation, native `Ohi`a and Koa mesic forest habitats, further suggesting that individuals within this genetic group might have been selected in this type of environment.

### 3.5 Signatures of local adaptation

**FIGURE 7:**
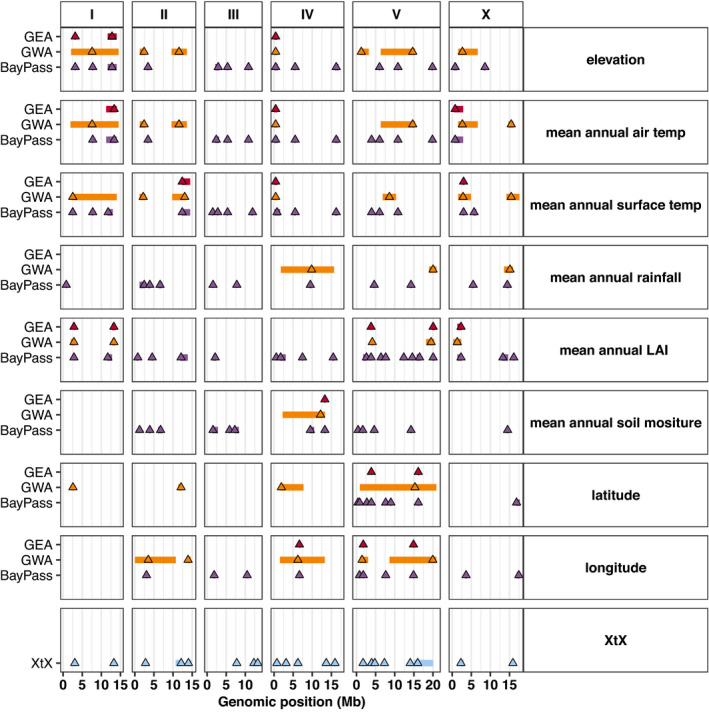
Genetic architecture of local adaptation. Genotype‐environment association (GEA) results for BayPass and GWA methods are plotted for eight environmental variables: elevation, mean annual air temperature, mean annual surface temperature, mean annual rainfall, mean annual leaf area index (LAI), mean annual soil moisture, latitude, and longitude. The BayPass XtX statistic is plotted on the bottom facet. Each triangle represents the marker with peak significance value within each region for each method. Regions are shown as rectangles plotted behind the peak marker position and their width is determined, as described in Materials and Methods. The peak markers and regions are coloured by their type (red, GEA region; orange, GWA region; purple, BayPass region; blue, XtX region). The GEA regions represent cases where BayPass, GWA, and XtX regions overlap. The overlap for BayPass and GWA are considered within the same environmental variable. If overlap exists, then the smaller of the two overlapping regions is compared to the XtX statistic. If the smaller of the two regions overlaps with the XtX statistic, then the region is determined to be a GEA region and plotted in red for each environmental variable. Genomic position is plotted along the x‐axis in megabases and by chromosome

Local adaptation can occur in response to different selection pressures caused by the environment. For instance, allele frequencies will correlate with selective features of the environment when the selection pressure can counteract homogenizing forces like migration (Haldane, 1948; Lenormand, 2002; Nagylaki, 1975). Genotype‐environment association (GEA) methods have the potential to detect these signatures of local adaptation and help elucidate their genetic basis in natural populations. We applied two separate GEA methodologies (BayPass and genome‐wide association, GWA) to detect signatures of local adaptation to various environmental parameters, including elevation, along with annual mean measures of ambient temperature, substrate temperature, rainfall, soil moisture, and leaf area index (see Materials and Methods). We also explored associations with latitude and longitude because selective pressures associated with geographic location could also underlie signatures of local adaptation. Using the NemaScan GWA pipeline (Widmayer et al., 2021), we found 39 regions of the genome that were associated with environmental or geographic parameters (Figure 7). The BayPass genome‐wide scan revealed 108 regions of the genome that were associated with environmental or geographic parameters (Figure 7). On average, the BayPass regions were smaller than the regions identified by GWA. We explored the consensus between these two GEA methods with respect to mappings for each environmental or geographic parameter and found a total of 35 regions that overlapped between the two methods. We refer to these 35 regions as method overlap regions. We also used the XtX statistics calculated by BayPass to scan for genomic regions that appeared to be adaptively differentiated when controlling for the covariance structure in population allele frequencies resulting from the demographic history of the populations (see Materials and Methods) (Gautier, 2015; Günther & Coop, 2013). This scan identified 21 regions of the genome consistent with adaptation to local environments. We then determined that 20 of the 35 method overlap regions fall into 11 XtX regions, we refer to these 20 regions as GEA regions (Figure 7). We prioritized these 20 GEA regions to explore the genetic basis of local adaptations to environmental parameters within the Hawaiian isotypes. We estimated the effect sizes for these regions by calculating the environmental variance explained at GWA markers with peak significance values (mean variance explained = 20.2%, *SD* = 16.1%, Figure S16). Among all the environmental parameters tested, mean annual air temperature and soil moisture have the highest cumulative variance explained (mean annual air temperature = 73%, mean annual leaf area index = 59%), which suggests that these variables are especially important drivers of local adaptation. In general, we observed that the genetic architectures of environmental associations were similar for correlated variables. For example, an identical region on chromosome IV at 0.8 megabases is associated with elevation and mean annual air and surface temperatures that have pairwise Pearson's correlation coefficients >0.95 or <–0.97 (Figure S3). Of the 20 GEA regions, 18 are distinct, because of the overlap on chromosome IV described above. Interestingly, all but one of the 18 distinct GEA regions fall into hyper‐divergent regions (Lee et al., 2021; Thompson et al., 2015). The hyper‐divergent regions are maintained by balancing selection and are enriched for genes related to environmental sensing, xenobiotic detoxification, and pathogen resistance. Therefore, the signatures of local adaptation that we observe in the Hawaiian strains match the expectation that these regions are important for the adaptation of *C*. *elegans* in its local environment.

## 4 DISCUSSION

This study represents the most detailed survey of Hawaiian nematodes to date. We isolated nematodes from five *Caenorhabditis* species and 18 other genera. Outside of *Caenorhabditis*, a number of nematode genera have been heavily studied, for example, *Oscheius*, *Pristionchus*, *Steinernema*, and *Heterorhabditis* (Campos‐Herrera et al., 2015; Sommer & McGaughran, 2013; Stock, 2015). We isolated many *Oscheius* but no *Pristionchus* or *Steinernema* nematodes. Interestingly, we did isolate entomopathogenic *Heterorhabditis* nematodes from two samples (Supporting Information 2). The presence of *Heterorhabditis* and absence of *Steinernema* is consistent with occurrence patterns of entomopathogenic nematodes on other tropical islands (Kour et al., 2020). Similar to previous studies, we found that Hawaiian *C*. *elegans* strains harbour high levels of genetic diversity relative to strains found in most other sampling locations, particularly locations in Europe (Andersen et al., 2012; Lee et al., 2021; Richaud et al., 2018; Rockman & Kruglyak, 2009). The *C*. *elegans* niche on the Hawaiian Islands is higher in elevation, cooler, drier, and less impacted by introduced plant communities and human disturbance than other selfing *Caenorhabditis* species. Importantly, our longitudinal sampling strategy uncovered multiple sites across various islands where *C*. *elegans* can be sampled reliably over time, enabling more detailed examinations of the temporal patterns of genetic variation in the species with additional sampling in the future. Furthermore, our quantitative measures of the *C*. *elegans* niche will allow for targeted exploration of new sampling locations on Hawaiian Islands and perhaps other Pacific Islands. In general, surveys of other islands in the Pacific and continental regions across the Pacific Rim will help contribute to our understanding of the evolutionary forces shaping the current patterns of genetic diversity.

### 4.1 Geographic patterns of genetic diversity

The enrichment of *C*. *elegans* in native habitats relative to introduced or disturbed habitats was one of the most striking patterns of genetic diversity that we found. By contrast, the other selfing species, *C*. *briggsae* and *C*. *tropicalis*, show the opposite enrichment pattern. These trends support the hypothesis that *C*. *elegans* is a native species to the Hawaiian Islands and also suggest that *C*. *briggsae* and *C*. *tropicalis* could be more recent invaders of the Hawaiian Islands. Other invasive taxa exhibit spatial distributions similar to *C*. *briggsae* and *C*. *tropicalis* (Jacobi et al., 2017). In many cases, these invasions are linked to human colonization and the environmental impacts humans impose on low elevation regions of the islands (Alison Kay, 1994). However, despite the enrichment patterns that we observe, further evidence is needed to determine whether *C*. *briggsae* and *C*. *tropicalis* are truly invasive to the Hawaiian Islands. To this end, genomic resources for the Hawaiian strains of these two species are currently under development. An alternative hypothesis for the differing niche enrichment patterns between these species could be that *C*. *briggsae* and *C*. *tropicalis* simply outcompete *C*. *elegans* at low elevation and *C*. *elegans* has been pushed to invade high elevation native habitats. Afterall, the temperatures at low elevation are more similar to the optimal temperatures for *C*. *briggsae* and *C*. *tropicalis* growth in the laboratory (Poullet et al., 2015). In regards to *C*. *elegans* as a native Hawaiian species, the progression rule predicts that differentiation within the species would follow the progression of island emergence as the Pacific Plate progresses over the Hawaiian hot spot (Shaw & Gillespie, 2016). The Hawaiian Islands are wholly volcanic and the islands we sampled progress in age along the west‐northwest direction; Big Island ~0.6 million years ago (Ma), Maui ~1.0 Ma, Molokai ~1.8 Ma, Oahu ~3.2 Ma, Kauai ~5.1 Ma (Neall & Trewick, 2008). Our genetic data partially support the notion of *C*. *elegans* as a native Hawaiian species conforming to the progression rule. Specifically, we found that the major axis of genetic variation in our Hawaiian sample (PC1) is most strongly correlated with the age of the islands. However, the trend is far from perfect as the distinct genetic groups that we identified do not correlate perfectly with island progression. For example, most groups are found distributed across multiple islands. However, the absence of perfectly associated progression patterns is not sufficient to rule out *C*. *elegans* as an early colonizer or native species to the Hawaiian Islands. Notably, other evolutionary forces like migration and selection may obscure patterns of diversity established by the progression of island formation. For example, more recent migration among the islands or from the mainland could be overlaid on the progression pattern. Indeed, evidence of gene flow between Hawaiian genetic groups and the west coast of North America was recently identified (Crombie et al., 2019). Moreover, we show here that the differentiation of genetic groups on the islands could also be influenced by heterozygous selection forces acting across the diverse habitats on the islands, as supported by the strong correlations that we observe between axes of genetic variation (PCs) and various environmental parameters as well as genomic scans detecting multiple signatures of local adaption.

Earlier studies have speculated that the Hawaiian Islands could be the geographic origin of the *C*. *elegans* species based on the high levels of genetic diversity observed on the islands relative to other sampling regions across the globe (Andersen et al., 2012; Crombie et al., 2019; Lee et al., 2021). Although our recent findings do not rule out this possibility, it is not definitive to suggest that the Hawaiian Islands are the geographic origin of the species. Many other regions around the Pacific Rim also harbour elevated levels of genetic diversity (Lee et al., 2021). Furthermore, far less sampling effort has been applied to those regions, suggesting that additional diversity is waiting to be discovered. For now, it is equally likely that the elevated diversity on the Hawaiian Islands could have been established through repeated colonization of the islands by a true origin along the Pacific Rim, possibly combined with founder effects that could further elevate diversity. Ultimately, species originating on volcanic islands are most convincingly inferred from sister groups on the same or neighbouring islands. However, despite our extensive sampling of the Hawaiian Islands, we have not identified a sister species there. *Caenorhabditis inopinata* remains the species most closely related to *C*. *elegans* and it was isolated from the Ishigaki Island in south Japan (Kanzaki et al., 2018). Further sampling of the Hawaiian Islands and especially around the Pacific Rim is needed before convincing inferences about the origin of this species can be made.

### 4.2 Other forces contributing to the genetic differentiation

In other selfing species such as *Arabidopsis thaliana*, regional patterns of differentiation have been attributed to isolation by distance (IBD) (Manzano‐Piedras et al., 2014). Genetically distinct groups are thought to have emerged largely because of dispersal limitation and genetic drift rather than geographically distinct selection pressures. The outsized role of stochastic processes evoked by IBD is similar to the strong influence of successive founder effects that drive differentiation under the progression rule. Under either model, we would expect to see a strong association between genetic distance and geographic distance across the Hawaiian Islands. However, our data deviate from the expectations of these models in two important ways. First, we see evidence of a single genetic group (orange) spread across nearly all sampling sites with no apparent geographic association. Second, another genetic group (purple) appears at just two sampling sites that are separated by over 500 km, nearly the maximum distance possible across our sampling sites. Dispersal distances for *C*. *elegans* have not been measured precisely, but numerous lines of evidence suggest they are greater than might be expected for such a small animal. By themselves, nematodes have been observed moving over relatively short distances, for example, 1 m in soil over a month period (Barrière & Félix, 2007). However, it was recently shown that wind‐mediated transport could facilitate long‐range dispersal for nematodes <0.75 mm in length, especially under high humidity conditions (Ptatscheck et al., 2018). *C. elegans* dauer larvae are typically 0.4 mm in length, desiccation resistant, and capable of surviving without feeding for months (Cassada & Russell, 1975; Klass & Hirsh, 1976). Furthermore, *C*. *elegans* has been isolated in association with several terrestrial invertebrate species, including snails, slugs, and isopods, which are thought to act as dispersal vectors and some molluscs can even survive passage through the guts of migratory birds (Petersen, Hermann, et al., 2015; Schulenburg & Félix, 2017; Wada et al., 2012). Finally, global patterns of genetic diversity in the species show that long‐range dispersal could be common given that many isotypes are sampled from locations at least 50 km apart (Lee et al., 2021). Notably, the isotype ECA251 could represent an extreme case of long‐range dispersal as it was isolated from locations that are over 13,000 km apart (California in 1973 and southern Australia in 1983). If the dispersal capacity of *C*. *elegans* is exceptionally large relative to the size of the Hawaiian Islands, then the genetic structure present on the islands could be shaped disproportionately by selection, especially given the extreme environmental heterogeneity found on the islands (Cutter, 2015). This scenario would be analogous to the Baas‐Becking hypothesis in microbial biogeography, which proposes “Everything is everywhere, but the environment selects’’ (Baas‐Becking, 1934; Finlay, 2002; Fuhrman, 2009). In other words, the distribution of microbes is theorized to occur in a regime of extensive dispersal and strong selection, generating local adaptation. Considering the disjointed geographic distribution of the purple group, we suspect that selection might also play a major role in shaping the geographic patterns of diversity on the islands. This idea is supported by the striking similarities in habitat and climate variables at the two sites where the purple group was sampled. Importantly, evidence of local adaptation does not rule out that neutral forces contribute or once contributed to the patterns of Hawaiian diversity because the two are not mutually exclusive. For example, recent studies of *A*. *thaliana* show that IBD and local adaptation each contribute to patterns of genetic differentiation at the regional scale on the Iberian peninsula (Castilla et al., 2020).

### 4.3 The genomic architecture of local adaptation

For reasons discussed above, population structure and complex demographic histories can mislead efforts to identify signatures of local adaptation. However, methods to make GEA more robust to various types of structure exist. In the case of a GEA using GWA, the NemaScan pipeline accounts for the relatedness and genetic structure among strains with a genomic relationship matrix (Widmayer et al., 2021; Yang et al., 2011). On the other hand, BayPass uses Bayesian hierarchical models to explicitly account for the scaled covariance matrix of population allele frequencies (Gautier, 2015). However, in either approach, the inferences made when using these tools are subject to limitations. For example, simulations testing the performance of the NemaScan pipeline with various mapping panels indicate that false discovery rates are higher when strongly differentiated strains are included in the mapping panel rather than more closely related strains (Widmayer et al., 2021). This limitation means that some regions implicated in local adaptation might reflect unresolved population structure among the sample of Hawaiian strains. A similar limitation applies to BayPass, wherein spurious signals of local adaptation to environmental variables could be caused simply by demography or drift rather than local adaptation. Conversely, corrections for structure can also reduce the power and increase the rate of false negatives in GEA, especially when major axes of genetic variation and selection coincide. Simulations indicate that higher rates of selfing causes greater neutral divergence between populations and can reduce the power of statistical methods to detect local adaptation loci (Hodgins & Yeaman, 2019).

Another consideration for GEA studies is that the environmental parameters measured might just be correlated with other selective parameters of the environment. In these cases, the true aspects of the environment imparting selective pressures cannot be known without further experiments. For example, suppose elevation gradients drove differences in microbial communities in Hawaiian habitats, as they do in other regions (Tang et al., 2020). Under this hypothetical scenario, differences in pathogenicity or nutritional quality of microbial communities might be the true driver of variation in selection pressure for Hawaiian *C*. *elegans* along elevation gradients.

In this study, we found that regions of the genome implicated in local adaptation to environmental parameters on the Hawaiian Islands frequently overlap with hyper‐divergent regions (Lee et al., 2021; Thompson et al., 2015). Hyper‐divergent regions are significantly enriched for genes that modulate sensory perception and responses to pathogens in wild habitats (Lee et al., 2021). For example, genomic loci overlapping with hyper‐divergent regions underlie natural variation in responses to the pathogens *Nematocida parisii* and Orsay virus (Ashe et al., 2013; Balla et al., 2015). For these reasons, we suspect that the selective pressures exerted by local microbial communities, although unmeasured in this study, might be especially important proximal drivers of local adaptation in *C*. *elegans*. In order to formally test this hypothesis, the microbial communities present at sampling sites should be preserved and characterized when possible.

Regardless of what factors appear to be driving patterns of local adaptation, candidate variants within putatively adaptive loci need to be identified and experimentally validated. Direct proof that a genetic variant causes a fitness advantage in a local environment can only be obtained experimentally (Rellstab et al., 2015), but these experiments are difficult to do in a laboratory setting. In plants, reciprocal transplant studies have been used to validate local adaptation to climate (Ågren & Schemske, 2012; Postma & Ågren, 2016). However, this design is challenging in *C*. *elegans* because methods for transplanting and resampling individuals have not been developed. As an alternative, validation experiments can be performed in the laboratory by exposing experimental populations to environmental extremes and recording allele frequencies for particular variants over time. This approach has been used successfully to validate fitness advantages of specific variants under anthelmintic drug exposure (Dilks et al., 2020, 2021; Hahnel et al., 2018) and to climate variables at collection sites (Evans et al., 2017). The disadvantage is that the complexity of the real environment is not recapitulated in the laboratory, and fitness advantages observed might not translate to natural conditions. The advantage is that the genetics and the environment can be exquisitely controlled in the laboratory. In the future, these validation techniques can probe whether candidate loci contain functional genetic variation that contributes to environmental adaptation.

## CONFLICTS OF INTERESTS

The authors have no conflicts of interest.

## AUTHOR CONTRIBUTIONS

Timothy A. Crombie and Erik C. Andersen designed the research. Timothy A. Crombie, Robyn E. Tanny, Kathryn S. Evans, Claire M. Buchanan, Daniel E. Cook, Clayton M. Dilks, Loraina A. Stinson, Daehan Lee, Stefan Zdraljevic, Gaotian Zhang, Nicole M. Roberto, Michael Ailion, and Erik C. Andersen performed the research. Timothy A. Crombie, Paul Battlay, and Kathryn S. Evans analysed the data. Timothy A. Crombie and Erik C. Andersen wrote and edited the manuscript.

## BENEFIT‐SHARING STATEMENT

Benefits from this research accrue from the sharing of our data and results on public databases as described above.

## Supporting information

Supplementary Material

Supplementary Material

Table S1‐S3

## Data Availability

All the data and code used to perform our analyses and figures have been made available for download at https://github.com/AndersenLab/molecular_ecology_manuscript, except for NemaScan code, which is available at https://github.com/AndersenLab/NemaScan. The variant sets used in our analyses are subset from the variant set released on 20210121 and available at https://www.elegansvariation.org/data/release/latest.
