# Supplementary material for: Local adaptation and spatiotemporal patterns of genetic diversity revealed by repeated sampling of Caenorhabditis elegans across the Hawaiian Islands
Source: Mol Ecol. 2022 Feb 25;31(8):2327–47. doi: 10.1111/mec.16400 (PMC9306471; doi:10.1111/mec.16400)
Supplement: Supplementary file 1 — Supplementary Material [file MEC-31-2327-s002.docx]

**Supplemental Information for:**

**Local adaptation and spatiotemporal patterns of genetic diversity revealed by repeated sampling of *Caenorhabditis elegans* across the Hawaiian Islands**

Timothy A. Crombie^1^, Paul Battlay^2^, Robyn E. Tanny^1^, Kathryn S. Evans^1^, Claire M. Buchanan^1^, Daniel E. Cook^1,3^, Clayton M. Dilks^1,3^, Loraina A. Stinson^1,3^, Stefan Zdraljevic^3^, Gaotian Zhang^1^, Dan Lu^1^, Nicole M. Roberto^1^, Daehan Lee^1^, Kathryn A. Hodgins^2^, and Erik C. Andersen^1,*^

1. Department of Molecular Biosciences, Northwestern University, Evanston, IL, USA

2. School of Biological Sciences, Monash University, Melbourne, Australia

3. Interdisciplinary Biological Sciences Program, Northwestern University, Evanston, IL, USA

**Table of Contents:**

| **Supplemental Figure 1** | Page 2 |
| --- | --- |
| **Supplemental Figure 2** | Page 3 |
| **Supplemental Figure 3** | Page 4 |
| **Supplemental Figure 4** | Page 5 |
| **Supplemental Figure 5** | Page 6 |
| **Supplemental Figure 6** | Page 7 |
| **Supplemental Figure 7** | Page 8 |
| **Supplemental Figure 8** | Page 9 |
| **Supplemental Figure 9** | Page 10 |
| **Supplemental Figure 10** | Page 11 |
| **Supplemental Figure 11** | Page 12 |
| **Supplemental Figure 12** | Page 13 |
| **Supplemental Figure 13** | Page 14 |
| **Supplemental Figure 14** | Page 15 |
| **Supplemental Figure 15** | Page 16 |
| **Supplemental Figure 16** | Page 17 |
| **Supplemental Table 1** | Page 18 |
| **Supplemental Table 2** | Page 19 |
| **Supplemental Table 3** | Page 20 |
| **Supplemental Files 1-18** | Page 21 |
| **Supplemental Files 19-25** | Page 22 |

**Supplemental Figure 1 - Cohabitation network of nematode taxa**

Cohabitation network with taxa as nodes. Node colors represent taxa (red is *C. elegans*, blue is *C. briggsae*, orange is *C. tropicalis*, other *Caenorhabditis* species are green, non-*Caenorhabditis* species are gray). The edges connecting nodes indicate the number of cohabitations between the nodes. The edge width corresponds to the number of cohabitations.

**Supplemental Figure 2 - Habitat enrichment for selfing *Caenorhabditis* nematodes**

The percentage of each sampling category is shown by habitat type. The sampling categories are colored according to the legend at the right, and the total number of samples for each substrate are shown on the right side of the bars. *C. elegans* are enriched in native habitats relative to introduced or distrubed habitats (Fisher’s Exact Tests, native vs. disturbed and native vs. introduced 𝘱 < 0.05 for both comparisons). *C. briggsae* are enriched in disturbed habitats relative to introduce or native habitats and enriched in introduced relative to native habitats (Fisher’s Exact Tests, disturbed vs. introduced, disturbed vs. native, and introduced vs. native, 𝘱 < 0.05 for all comparisons). *C. tropicalis* is enriched in disturbed habitats relative to native habitats, (Fisher’s Exact Test, native vs. disturbed 𝘱 > 0.05). All enrichment tests for selfing *Caenorhabditis* strains not listed were not significant (Bonferroni adjusted 𝘱 values ≥ 0.05).

**Supplemental Figure 3 - Environmental parameter correlations**

A correlation matrix for the continuous environmental parameters is shown. The parameter labels for the matrix are printed on the diagonal, and the Pearson correlation coefficients are printed in the cells. The color scale indicates the strength and sign of the correlations shown in the matrix.

**Supplemental Figure 4 - Environmental parameters for selfing *Caenorhabditis* collections**

Environmental parameter values measured at the time of collection: elevation (A), ambient temperature (B), substrate temperature (C), and ambient humidity (D). Environmental parameter values obtained from environmental models: mean annual air temperature (E), mean annual surface temperature (F), mean annual precipitation (G), mean annual available soil moisture (H), mean annual leaf area index (I). Tukey box plots are plotted by species (red = *C. elegans*, orange = *C. tropicalis*, blue = *C. briggsae*) for each environmental parameter. Letters above the boxes summarize statistical significance of comparisons between the species shown. Species with a different letter are significantly different; species with the same letter are not different. Comparisons were made using a Kruskal-Wallis test and Dunn’s test for multiple comparisons with 𝘱-values adjusted using the Bonferroni method.

**Supplemental Figure 5 - Substrate sampling bias by habitat status**

The percentages of collections within each habitat class are plotted by substrate type. Each of the three habitat classes are shown as vertical bars and colored as indicated in the key on the right. Each facet represents a unique substrate type and is labeled on top of the plot. For each habitat class, the bars across all facets sum to 100%.

**Supplemental Figure 6 - Substrate sampling bias by land cover.**

The frequencies of collections within each land cover are plotted by habitat class and substrate. The three habitat classes are plotted in (A) native habitat, (B) introduced habitat, and (C) disturbed habitat. Within each habitat class, land cover types are plotted on the x-axis and colored as indicated in the key on the right. The facets represent unique substrate types and are labeled on the top of the plot.

**Supplemental Figure 7 - Geographic distance between isotype samples**

The histogram shows the counts of pairwise Haversine distances between samples within an isotype. Haversine distance is calculated as the angular distance between two points on the surface of a sphere and is used to account for the curvature of the Earth. Counts are on the y-axis, and physical distance between samples is on the x-axis. The dashed red line indicates 500 meters, only four pairwise distances exceed 500 meters. The collection date and strain name for each pair isolated over 500 meters from each other are shown in the boxes separated by a colon. The isotype for each strain pair is also shown on the second line within the box.

**
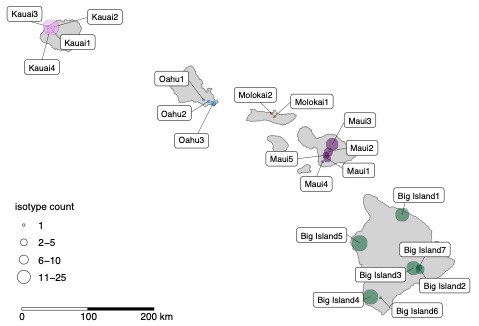
**

**Supplemental Figure 8 - Map of three kilometer sampling locations for *C. elegans***

The 21 three kilometer diameter sampling locations are plotted on a map of the Hawaiian Islands. The center of each circle indicates the centroid of the sampling location. The size of the circle represents the number of isotypes found within the location. We sampled a single isotype from seven locations. The labels indicate the names of each location and are connected to the centroid by a black line. The circles are colored by island.

**Supplemental Figure 9 - Temporally persistent isotypes by sampling location**

Sample frequencies for temporally persistent isotypes are plotted by collection time for each sampling location: (A) Big Island 1, (B) Big Island 3, (C) Big Island 5, (D) Maui 1, (E) Maui 2. Within each sampling location, the persistent isotypes are plotted with a unique color corresponding to the key to the right of the plot. Non-persistent isotypes are always plotted in gray. The total number of isotypes sampled for each collection time is shown above the bars. In B and E, the 2016-Feb time point refers to a small sampling effort that we did not include as one of the six major sampling efforts.

**Supplemental Figure 10 - Sampling frequency of *Caenorhabditis* species and preferred substrates by month**

(A) The sampling frequencies of *Caenorhabditis* species by sampling month. The bars are colored by species. For *C. elegans*, sampling frequency is lowest in August, intermediate in October, and highest in December, (Fisher’s Exact Tests, 𝘱 < 0.05 for all comparisons). Other species do not exhibit a seasonal trend. (B) Sampling frequencies of preferred substrates by month. Fruit and flower substrates are considered “preferred”, all other substrate types are considered “other”. Preferred substrates were sampled more frequently in December relative to August or October (Fisher’s Exact Tests, 𝘱 < 0.05).

**
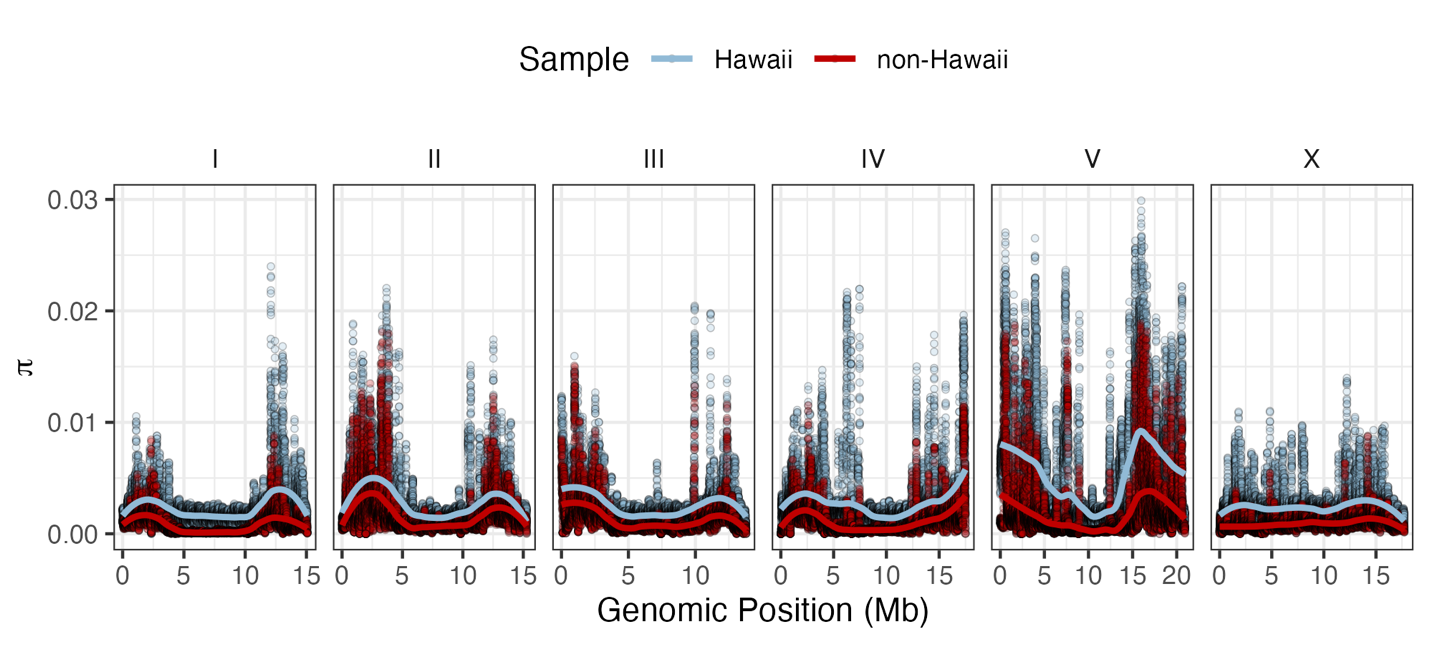
**

**Supplemental Figure 11 - Genome-wide pi for Hawaiian and non-Hawaiian samples**

(A) Nucleotide diversity (pi) measured across the genome for 163 Hawaiian isotypes (blue) and 377 non-Hawaiian isotypes (red) is shown. Genomic position is on the x-axis, and facets indicate chromosomes. Genome-wide pi was calculated along sliding windows with a 10 kb window size and a 1 kb step size.

**
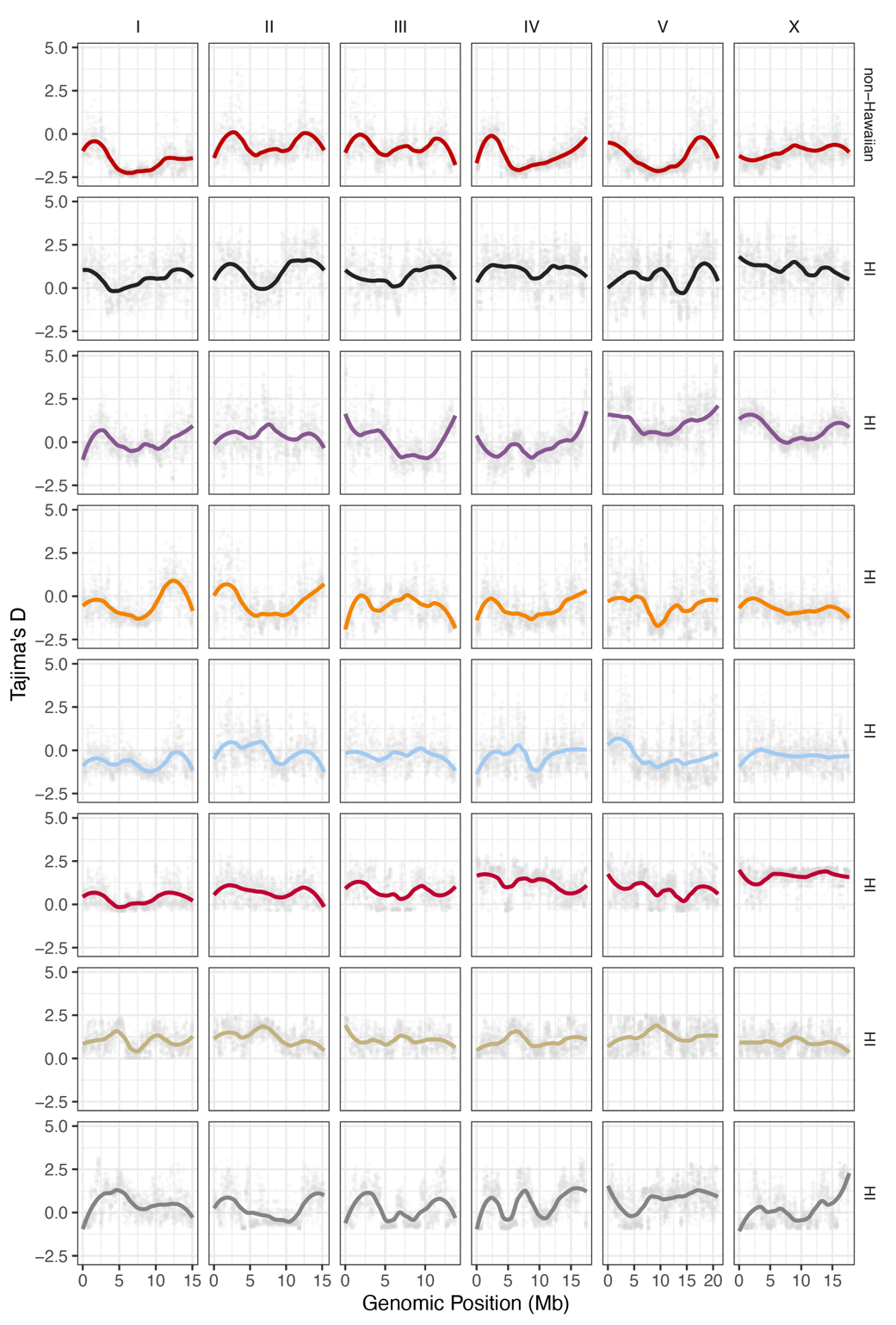
**

**Supplemental Figure 12 - Genome-wide Tajima’s D for Hawaiian and non-Hawaiian samples**

Tajima’s D is measured across the genome for 377 non-Hawaiian isotypes (red, top horizontal facet labeled “non-Hawaiian”) and for the isotypes in each of the Hawaiian genetic groups (facets labeled “HI”, colored by group assignment). Genomic position is on the x-axis and vertical facets indicate chromosomes. Genome-wide Tajima’s D was calculated for 10 kb windows.

**Supplemental Figure 13 - *Caenorhabditis elegans* genetic relatedness**

An unrooted tree built with single nucleotide variants found among the 540 *C. elegans* isotypes sampled from the wild (see Materials and Methods). The branch tips of isotypes sampled from the Hawaiian Islands are shown with a circle (163), and isotypes sampled from other regions are shown with a triangle (377). For each isotype, we classified chromosomes I, IV, V, and X as swept if ≥ 30% of the chromosome consisted of the swept haplotype. Branch tips are colored by the number of swept chromosomes identified for that isotype. The labeled isotypes are either non-Hawaiian isotypes that group with divergent Hawaiian isotypes (ECA2581, ECA36, QX1211, JU3226), or Hawaiian isotypes that contain one or more swept chromosomes (ECA928, ECA923, QX1792, ECA1413, ECA2111, XZ1515). The labels for isotypes are colored by the number of swept chromosomes.

**Supplemental Figure 14 - Population structure by principal components analysis**

Bi-plots of significant principal components of genetic variation for 163 Hawaiian isotypes. Points represent distinct isotypes, and the axes correspond to values for principal components (PCs) labeled on the top and right of the plots. The plots below the diagonal (open white boxes) show PC values calculated for all 163 isotypes, and the plots above the diagonal show PC values calculated for 149 non-outlier isotypes. **A**) Bi-plots with isotypes colored by island of isolation. **B**) Bi-plots with isotypes colored by genetic clusters revealed by PCA of genetic variation for 149 non-outlier isotypes.

**Supplemental Figure 15 - Effect of linkage disequilibrium pruning on population structure inference**

**A-D**) Each tree represents the genetic relatedness of the 163 Hawaiian isotypes. The trees are constructed with whole-genome SNV data with varying levels of linkage disequilibrium (LD) pruning thresholds (*r^2^* = 0.8, 0.6, 0.2, and 0.1). Branch tips are colored according to the genetic group to which they are assigned by PCA. The number of isotypes assigned to groups is shown above each plot.

**Supplemental Figure 16 - Environmental parameter values by genotype at peak GWA markers**

Environmental parameter values for isotypes are plotted by genotype at marker positions with maximal significance in GWA. The 18 plots represent the peak marker positions for every GWA region overlapping with GEA regions in Figure 7. The peak marker positions and environmental parameter variance explained at that position are shown above the plot. The environmental parameter values are on the y-axis, genotype is on the x-axis, and data points represent median parameter values for each isotype.

**Supplemental Table 1 - Distinct collection type counts by project**

Each collection class is shown by row and each collection project is shown by column. Counts in each field represent distinct substrates for which a collection class was isolated. In many cases, more than one collection type was isolated from a single substrate so the totals are greater than the actual number of substrates collected (4,506).

**Supplemental Table 2 - Niche variation among isotypes sampled from multiple substrates**

The environmental niche parameters for the 31 isotypes sampled from multiple substrates are shown. For categorical variables, unique values within the isotype are shown. For continuous variables, the values are summarized if more than three substrates were sampled. The first value represents the median and the values within the parentheses indicate the range. Missing data are represented by either NA or no value.

**Supplemental Table 3 - *C. elegans* sampling clusters**

The 21 three-kilometer sampling clusters are provided as rows. Sampling information relating to *C. elegans* isolates are provided in columns. The “samples” column shows the number of *C. elegans* positive samples found within the cluster. The “isolates” column shows the number of *C. elegans* isolated from samples in the cluster. In some cases, the “strains” column is lower than the “isolates” column because isolates were lost before they could be cryopreserved and whole-genome sequenced.

**Supplemental File 1** A list of all cryopreserved strains presented in this study.

**Supplemental File 2** Sampling data for every Hawaiian collection used to generate Figure 1.

**Supplemental File 3** Collection class frequencies for each land cover type within habitat class used to generate Figure 2.

**Supplemental File 4** Collection class frequencies for each substrate type used to generate Figure 3A.

**Supplemental File 5** Environmental parameter values for samples used to generate Figure 3B-G, Supplemental Figure 3, and Supplemental Figure 4.

**Supplemental File 6** Genetic group assignments and PC values for all non-outlier isotypes used to make Figure 4A-B, Supplemental Figure 14, and Supplemental Figure 15D.

**Supplemental File 7** Geographic locations of sampling sites for isotypes with genetic group assignments used to make Figure 4C.

**Supplemental File 8** Continuous environmental parameter values for distinct collections within each genetic group used to generate Figure 5.

**Supplemental File 9** Correlation matrix for significant genetic PCs by continuous environmental parameters and island age used to make Figure 6.

**Supplemental File 10** BayPass XtX statistics for SNVs used to generate Figure 7.

**Supplemental File 11** GWA regions used to generate Figure 7.

Supplemental File 12 GEA regions used to generate Figure 7.

**Supplemental File 13** Counts for collection classes by project used to generate Supplemental Table 1.

**Supplemental File 14** Cohabitation counts for distinct taxa used to generate Supplemental Figure 1.

**Supplemental File 15** Sampling frequencies for habitat classes by substrate type used to generate Supplemental Figure 5.

**Supplemental File 16** Sampling frequencies for land cover class by substrate type grouped by habitat class used to generate Supplemental Figure 6.

**Supplemental File 17** Pairwise geographic distances between distinct collections within each isotype used to generate Supplemental Figure 7.

**Supplemental File 18** Geographic coordinates of 3 km diameter sampling clusters and the number of isotypes sampled within each cluster used to generate Supplemental Figure 8.

**Supplemental File 19** Sampling frequencies of temporally persistent isotypes over time for each sampling location used to generate Supplemental Figure 9.

**Supplemental File 20** Genome-wide pi calculated for 163 Hawaiian and 377 non-Hawaiian isotypes in sliding 10 kb windows that was used to generate Supplemental Figure 11.

**Supplemental File 21** Genome-wide Tajima’s D calculated for 163 Hawaiian and 377 non-Hawaiian isotypes in 10 kb windows that was used to generate Supplemental Figure 12.

**Supplemental File 22** Sweep status and number of swept chromosomes for 540 isotypes sampled from around the world used to generate Supplemental Figure 13.

**Supplemental File 23** PC values for all 163 Hawaiian isotypes generated by PCA without outlier removal that was used to generate Supplemental Figure 14.

**Supplemental File 24** Genetic group assignments derived from PCA and clustering using different LD thresholds prior to PCA (*r*^2^ = 0.8, 0.6, 0.2, 0.1) used to make Supplemental Figure 15.

**Supplemental File 25** Processed mapping data from GWA for each GWA peak marker overlapping with GEA regions used to make Supplemental Figure 16.
